# Hiperpotasemia mantenida en Atención Primaria en una paciente sin sintomatología de interés. ¿Cuándo sospechar de una pseudohiperpotasemia familiar?

**DOI:** 10.1515/almed-2022-0004

**Published:** 2022-07-11

**Authors:** Carlos Castillo Pérez, Laura Rodríguez Alonso, Adrián Prados Boluda, Marta Cebrián Ballesteros, Blanca Torrubia Dodero

**Affiliations:** Médico adjunto de Análisis Clínicos. Departamento de bioquímica general, hormonas y marcadores tumorales. Servicio de Bioquímica Clínica, Hospital Universitario Fundación Jiménez-Díaz, Madrid, España; Química adjunta de Análisis Clínicos. Departamento de bioquímica general, hormonas y marcadores tumorales. Servicio de Bioquímica Clínica, Hospital Universitario Fundación Jiménez-Díaz, Madrid, España; Médico residente de segundo año. Departamento de bioquímica general, hormonas y marcadores tumorales. Servicio de Bioquímica Clínica, Hospital Universitario Fundación Jiménez-Díaz, Madrid, España; Farmacéutica adjunta de Análisis Clínicos. Departamento de bioquímica general, hormonas y marcadores tumorales. Servicio de Bioquímica Clínica, Hospital Universitario Fundación Jiménez-Díaz, Madrid, España

**Keywords:** incubación, potasio, pseudohiperpotasemia

## Abstract

**Objetivos:**

El estudio y abordaje de un caso clínico de una paciente con elevaciones de potasio en las sucesivas revisiones sin justificación clínica.

**Caso clínico:**

Presentamos un caso clínico de una paciente con una elevación de potasio en las sucesivas analíticas realizadas por su médico de Atención Primaria, sin justificación clínica. La paciente es derivada a Nefrología, con potasemias mantenidas de 5,3–5,9 mmol/L en suero sin más datos de interés, tratada con dieta baja en potasio sin éxito. En el estudio completo no se encuentra ninguna causa orgánica que justifique la elevación.

**Conclusiones:**

Ante resultados discordantes entre las cifras de potasio y el motivo de consulta, y tras haber descartado otras causas preanalíticas o patológicas, se propone que la posible causa sea una pseudohiperpotasemia familiar. Se realiza un protocolo de incubación de la muestra a diferentes tiempos y temperaturas para demostrar su influencia en los niveles de potasio en sangre, por lo que se concluye el diagnóstico más probable de pseudohiperpotasemia familiar. Finalmente, la paciente es dada de alta por parte de Nefrología, con lo que puede retomar una dieta normal.

## Introducción

La elevación del potasio puede conllevar a situaciones clínicas desfavorables para el paciente, tales como arritmias cardíacas, debilidad muscular o parálisis [1]. Debido a ello, los laboratorios deben asegurar un correcto control de calidad preanalítico y analítico para el diagnóstico y seguimiento de las patologías derivadas del potasio.

**Figura 1:**
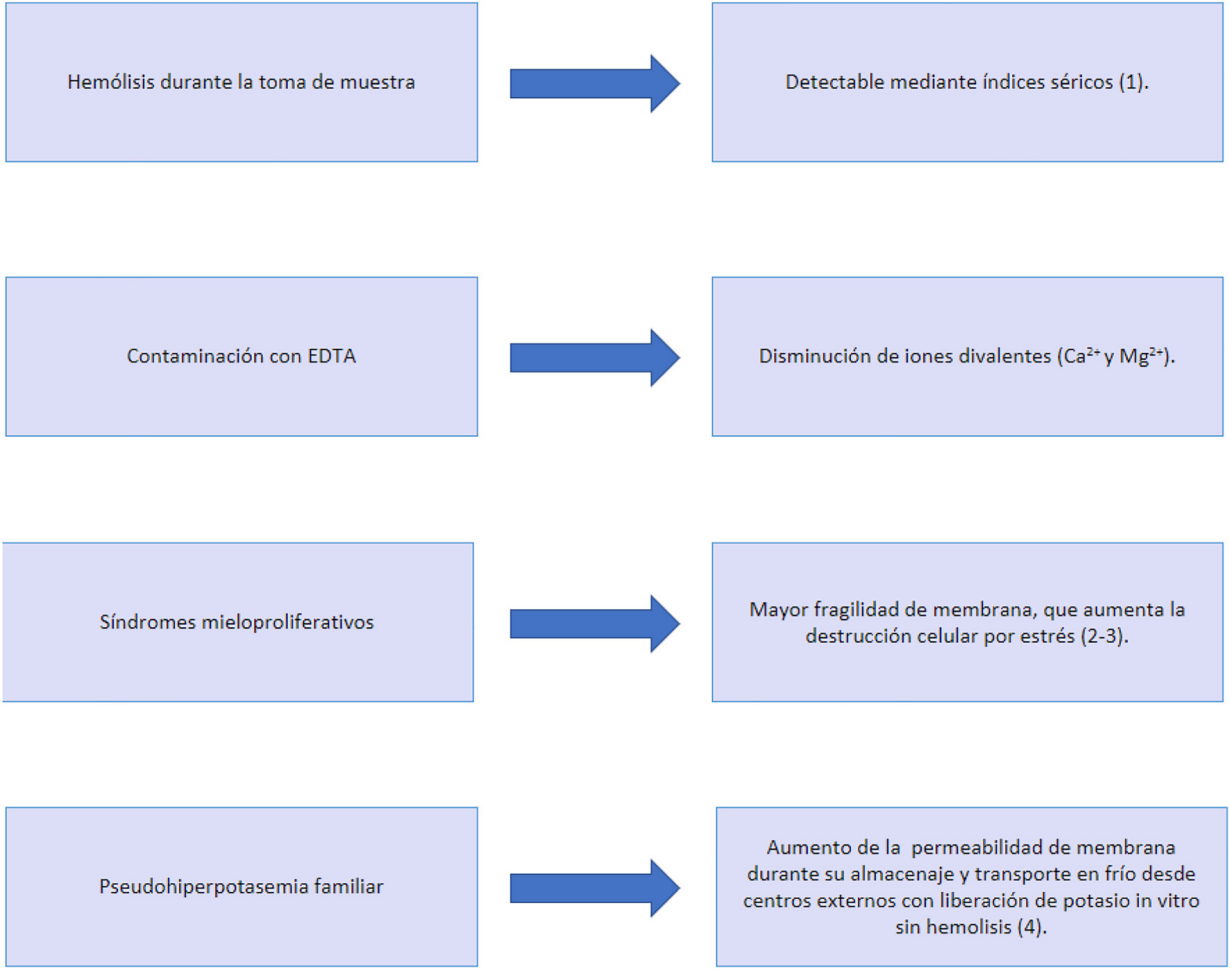
Causas más frecuentes de pseudohiperpotasemia.

La pseudohiperpotasemia se define como una elevación del potasio *in vitro*, que no se corresponde con los niveles reales en el organismo. Se describen las causas de pseudohiperpotasemia en la Figura 1.

Existe también una entidad clínica conocida como pseudohiperpotasemia familiar (PF). En esta, se produce una elevación de potasio *in vitro* debido al aumento de permeabilidad de la membrana del hematíe por la conservación a 4 °C.

Es importante que los laboratorios aseguren una correcta estrategia para detectar aquellos casos en los que se produzca una elevación de potasio en pacientes, sin aparente concordancia con la situación clínica, descartando las causas más frecuentes de pseudohiperpotasemia.

## Caso clínico

Paciente mujer de 57 años en seguimiento por su médico de Atención Primaria por una hiperpotasemia leve persistente de 4 años de evolución [rango 5,3–5,9 mmol/L (valor de referencia: 3,5–5,1 mmol/L)] sin presentar hemólisis (índice de hemólisis espectrofotométrico <6 mg/dL en todos sus informes). La paciente no presenta ninguna sintomatología relacionada ni ningún otro dato analítico de interés. Los sucesivos electrocardiogramas realizados son normales, sin hallazgos significativos. Como antecedentes personales, presenta una hipertensión arterial con buen control con IECAs. Debido a ello, la paciente es derivada a Nefrología para su estudio.

En la consulta de Nefrología, la anamnesis y la exploración física no revelaron datos de interés; se encuentra en tratamiento con amlodipino para la hipertensión arterial, con buen control. Se repite el electrocardiograma, que sigue siendo normal. Se pauta tratamiento dietético pobre en potasio y control analítico en 3 meses. Una vez pasados los 3 meses, la paciente acude a su centro de salud para realizar la extracción de control. En el laboratorio se recibe una muestra de suero con solo petición para potasio. La muestra no presenta hemólisis (índice de hemólisis <6 mg/dL) con una leve hiperpotasemia de 5,5 mmol/L. Desde el servicio de Nefrología plantean el siguiente diagnóstico diferencial una vez descartada una causa preanalítica y de medicación que pudiese causar hiperpotasemia:

1. Hiperpotasemia por insuficiencia renal.
2. Hiperpotasemia por hemólisis intravascular.
3. Hiperpotasemia por acidosis.
4. Hiperpotasemia por liberación tisular (rabdomiólisis).
5. Pseudohiperpotasemia por enfermedades oncohematológicas (trombocitosis esencial, leucemias agudas o crónicas).

Para realizar el diagnóstico diferencial desde el punto de vista analítico, se solicita que la extracción de sangre se lleve a cabo en la sala de extracciones del hospital con las siguientes pruebas:

– Hemograma con reticulocitos y frotis de sangre.
– Bioquímica general con iones, índice de hemólisis, perfil hepático (GOT, GPT, GGT, LDH, ALP), perfil renal (creatinina, urea y filtrado glomerular estimado CKD-EPI), creatin-quinasa, haptoglobina, metabolismo fosfocálcico y del hierro (hierro, índice de saturación de la transferrina, transferrina y ferritina).
– Gasometría venosa. La gasometría se procesa de manera urgente con un pH de 7,39 (Valor de referencia 7,35–7,45).
– Bioquímica en orina de 24 horas.

Se recibe una muestra de EDTA para el hemograma y una muestra de heparina de litio para la bioquímica. El frotis es normal, sin alteraciones morfológicas. El hemograma se encuentra dentro del rango de referencia en todos los parámetros a estudio con unos reticulocitos de 1,55% (valor referencia: 0,9–2,6%). Se descarta así en el diagnóstico diferencial la presencia de enfermedades oncohematológicas.

En la bioquímica general solo se observa alterado un colesterol de 248 mg/dL (valor de referencia: <200 mg/dL), con un potasio de 3,98 mmol/L (valor de referencia en plasma: 3,4–4,5 mmol/L), una creatinina de 0,86 mg/dL (valor de referencia: 0,51–0,95 mg/dL) y una haptoglobina de 39 mg/dL (valor de referencia: 30–200 mg/dL), y con un índice de hemólisis <6 mg/dL. La PCR, CK, perfil hepático y demás parámetros analizados se encuentran dentro del rango de referencia. Con estos datos se concluye que la paciente no presenta hiperpotasemia en el análisis realizado, sin insuficiencia renal ni datos de lisis celular y sin signos de hemólisis intravascular. La bioquímica de orina es normal, sin ningún valor fuera del rango de normalidad.

Una vez descartadas las causas mencionadas en el diagnóstico diferencial, junto a la ausencia en esta extracción de un potasio elevado, se valora un posible caso de PF. Se cita a la paciente para la extracción de 3 tubos de heparina de litio en el propio hospital para la realización de un protocolo de incubaciones a diferentes tiempos y temperaturas:

1. Dos alícuotas para las incubaciones a 4 °C durante 2 horas y 4 horas respectivamente. De esta manera se simulan las condiciones de transporte de las muestras desde los centros de atención primaria al laboratorio, ya que son transportadas a 4 °C en un tiempo medio de 3 horas.
2. Dos alícuotas para las incubaciones a 25 °C durante 2 horas y 4 horas respectivamente. De esta manera se simulan las condiciones de transporte de las muestras desde la sala de extracciones del hospital al laboratorio, ya que son transportadas a temperatura ambiente en un tiempo medio de 3 horas.
3. Dos alícuotas para las incubaciones a 37 °C durante 2 horas y 4 horas respectivamente. De esta manera se simulan las condiciones *in vivo*.

**Figura 2:**
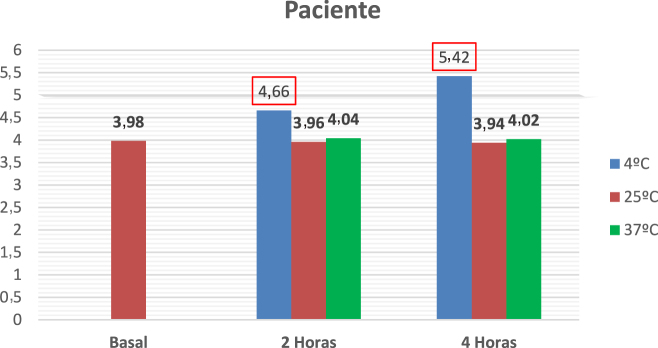
Muestra del paciente. Resultados de potasio en la muestra de la paciente tras las incubaciones a diferentes tiempos y temperaturas. Rojo: valores por encima del rango de referencia.

**Figura 3:**
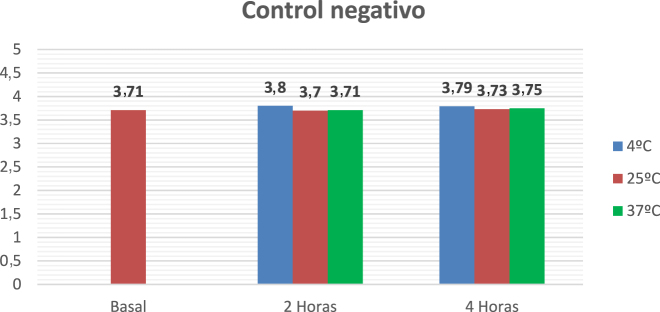
Muestra control negativo. Resultados de potasio tras incubaciones a diferentes tiempos y temperaturas.

Se reciben las muestras sin centrifugar para la realización del protocolo. La centrifugación se realiza una vez acabado el tiempo de incubación. Los resultados se muestran en la Figura 2. Se lleva a cabo un protocolo de un control negativo (los resultados se muestran en la Figura 3).

## Discusión

Las alteraciones electrocardiográficas y las neurológicas son las primeras manifestaciones de la hiperpotasemia [2]. Estas, son debidas a un aumento de la excitabilidad tisular, lo que puede conllevar a la aparición de arritmias malignas (taquicardias complejas o fibrilación ventricular) y neurológicas (calambres, parálisis o parestesias) [2]. Estas alteraciones muestran una buena correlación entre la concentración de potasio y los cambios producidos en el electrocardiograma [3].

En esta paciente, se descartaron las principales causas orgánicas de hiperpotasemia, no encontrándose ninguna alteración analítica. Además, no presentó hemólisis en ninguna muestra, ni otras causas menos frecuentes de pseudohiperpotasemia, como lo son los síndromes oncohematológicos. El hallazgo de una concentración plasmática de potasio normal cuando se realizó la extracción en el hospital, comparado con los potasios elevados cuando se transportaban a 4 °C desde Atención Primaria, hizo sospechar una posible PF.

**Figura 4:**
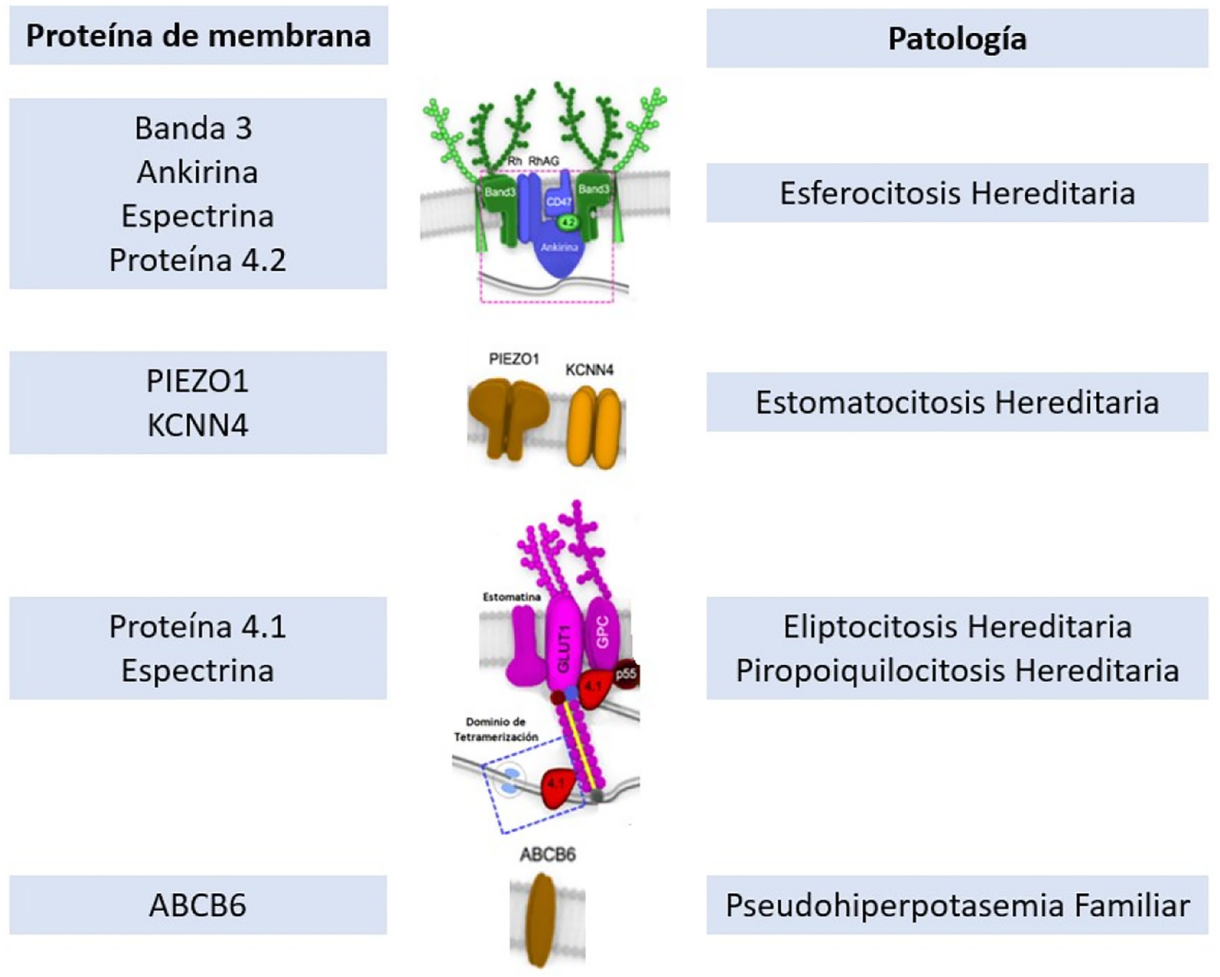
Representación esquemática de las proteínas de membrana del hematíe con sus patologías asociadas. Basada en: Yonggoo K, Joonhong P, Myungshin K. Diagnostic approaches for inherited hemolytic anemia in the genetic era. Blood Res. 2017 Jun; 52 (2):84–94.

La PF se encuadra dentro de las membranopatías eritrocitarias. Dentro de las membranopatías podemos diferenciar (Figura 4):

1. Anemia hemolítica por defectos de la membrana sin alteraciones de la permeabilidad. La esferocitosis, la piropoiquilocitosis y la eliptocitosis hereditaria forman parte de este grupo.
2. Anemia hemolítica por defectos de la membrana con alteraciones de la permeabilidad. La estomatocitosis hereditaria es un ejemplo de este grupo.
3. Defectos de la membrana con alteraciones de la permeabilidad sin anemia. La PF forma parte de este grupo.

La PF se produce por una mutación en la región 2q35–q36 en el gen ABCB6 con herencia autosómica dominante [4]. Es infrecuente observar alteraciones morfológicas en el frotis. En la PF se produce una liberación de potasio a bajas temperaturas (por debajo de 8-10 °C), con resultados normales a temperaturas superiores a 25 °C [5]. La proteína ABCB6 es una ATPasa de membrana inicialmente identificada como un transportador de porfirinas, aunque su función actualmente no está del todo esclarecida. Las mutaciones conocidas, no producen una disminución en la síntesis, sino una ganancia en la función del transportador [5]. Esto conlleva a una pérdida de potasio a bajas temperaturas.

Es importante aconsejar a estos pacientes no ser donantes de sangre. La conservación de las bolsas de concentrado de hematíes en neveras produce la liberación de potasio. Existen casos descritos con eventos adversos incluso muerte tras la transfusión de bolsas de pacientes con PF [6–8].

La paciente fue derivada a Nefrología por cifras de potasio repetidamente elevadas, sin una clínica que lo justificara. Se ofreció una terapia de restricción de alimentos ricos en potasio sin éxito analítico. En los casos en los que esta terapia fracasa y sin causa orgánica, se debe sospechar la presencia de una PF. La realización del ensayo mediante incubaciones de las alícuotas nos permite simular los diferentes escenarios preanalíticos a los que nos enfrentamos desde los centros de referencia. De esta forma, podemos comparar el comportamiento *in vitro* de la permeabilidad de los hematíes y determinar si la causa de la hiperpotasemia es debida a una probable PF. Para un diagnóstico definitivo, sería aconsejable la secuenciación del gen ABCB6 para la detección de las mutaciones conocidas para esta entidad.

## Conclusiones

En aquellos casos en los que la situación clínica del paciente no concuerde con un potasio elevado y/o tras indicaciones y tratamientos para bajar la potasemia sin resultado, se debe contactar con el laboratorio clínico para el estudio de la hiperpotasemia mediante diferentes abordajes, tanto clínicos como metodológicos que permitan el despistaje de una PF. De ello, depende la efectividad o no de los tratamientos, así como la calidad de vida de nuestros pacientes.
